# Underwater microscopy for *in situ* studies of benthic ecosystems

**DOI:** 10.1038/ncomms12093

**Published:** 2016-07-12

**Authors:** Andrew D. Mullen, Tali Treibitz, Paul L. D. Roberts, Emily L. A. Kelly, Rael Horwitz, Jennifer E. Smith, Jules S. Jaffe

**Affiliations:** 1 Scripps Institution of Oceanography, University of California San Diego, 9500 Gilman Drive, La Jolla, California 92093, USA; 2 School of Marine Sciences, University of Haifa, Haifa 3498838, Israel; 3 The Mina and Everard Goodman Faculty of Life Sciences, Bar-Ilan University, Ramat-Gan 52900, Israel; 4 The Interuniversity Institute for Marine Sciences, Eilat 88103, Israel

## Abstract

Microscopic-scale processes significantly influence benthic marine ecosystems such as coral reefs and kelp forests. Due to the ocean's complex and dynamic nature, it is most informative to study these processes in the natural environment yet it is inherently difficult. Here we present a system capable of non-invasively imaging seafloor environments and organisms *in situ* at nearly micrometre resolution. We overcome the challenges of underwater microscopy through the use of a long working distance microscopic objective, an electrically tunable lens and focused reflectance illumination. The diver-deployed instrument permits studies of both spatial and temporal processes such as the algal colonization and overgrowth of bleaching corals, as well as coral polyp behaviour and interspecific competition. By enabling *in situ* observations at previously unattainable scales, this instrument can provide important new insights into micro-scale processes in benthic ecosystems that shape observed patterns at much larger scales.

## Abstract (web summary)

Underwater microscopes have limited spatial and temporal resolutions. Here, Mullen *et al*. present a small non-invasive underwater microscope for both direct and fluorescence microscopy. They image coral bleaching and interspecific competition with resolutions approaching a micron and hundreds of milliseconds.

## Graphical abstract

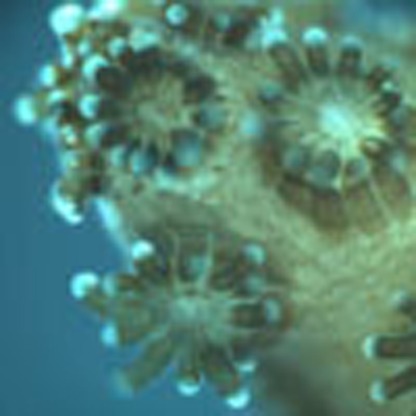

The health and long-term dynamics of coastal ecosystems such as kelp forests, mangroves, seagrass beds and coral reefs are significantly impacted by activities that occur on scales of a millimetre or less. Examples of these activities include: coral bleaching (expulsion of single-celled symbiotic algae)^1^, larval attachment and survival^2^, competition between organisms along thin interfaces^3^,^4^,^5^, and fluxes of particles to and from the seafloor^6^,^7^. These and other small-scale marine processes are of interest to scientists across diverse disciplines such as physiology, ecology, biomechanics and geology. However, in studying these systems it is important to consider that coastal oceans are complex and dynamic. The physical environment is continuously changing with high spatial and temporal variations in oxygen, pH^8^, temperature and fluid motion^9^. Organisms interact with each other in complex and non-linear ways across a wide range of scales^10^. In addition, many of these biological and physical processes are coupled and influence one another^8^,^9^. It is therefore difficult to study these processes in the lab, as it is impossible to fully replicate the intricacies of these natural systems *in vitro*. As a result, a distinct need exists to make observations of important environmental processes *in situ*, under natural conditions.

Previous calls for technology to perform micro-scale imaging in the ocean include that of Smetacek^11^ who asked ‘Could such an instrument (an *in situ* computerized telemicroscope) do for microbial ecology what Galileo's telescope did for astronomy?'. Underwater optical systems have been developed to image seafloor environments at large scales^12^,^13^, as well as to image free-floating zooplankton and larger phytoplankton at millimetre scales^13^,^14^. In addition, holography^15^ has been used to acquire microscopic images of free-floating plankton. However, the development of underwater microscopes to image benthic seafloor organisms at close to micrometre resolution has been lacking^13^,^14^. Imaging the seafloor environment presents significant additional challenges including active instrument positioning, precise focusing and reflectance illumination. Previous efforts to observe microscopic activities on the marine benthos have been significantly constrained in their potential applications due to both their highly intrusive nature and limited ability to collect spatial and temporal data^16^,^17^,^18^. A fundamental deficiency thus exists in our ability to observe micro-scale biological and environmental processes as they naturally occur near the seafloor. This hinders our capacity to connect conceptual models and lab studies to real world environments. It also impedes efforts to identify underlying mechanisms that drive large scale ecosystem change^10^,^19^,^20^.

To address these observational needs, we developed the Benthic Underwater Microscope (BUM). The BUM is an imaging system that provides the first *in situ*, underwater observations of benthic environments at nearly micrometre resolution. The diver-deployed, portable instrument can record dynamic natural processes and spatial patterns with minimal disturbance to benthic organisms or their surrounding physical environment. In addition, extended time-series recordings can be collected to reveal slow or periodic activities and processes, allowing for studies of animal behaviour (for example, individual coral polyps). These capabilities allow both temporal and spatial analysis of ecologically significant phenomena at scales never before seen in the natural environment. Here we detail the instrument's novel design, which addresses the unique challenges of underwater microscopy. We then demonstrate the system's capabilities with *in situ* images and videos of reef building corals. This includes time-series observations of coral behaviour and competition, as well as a quantitative analysis of *in situ* algal colonization patterns on bleached coral tissue during the 2015 coral bleaching event in the Main Hawaiian Islands.

## Results

### Instrument design

**Figure 1:**
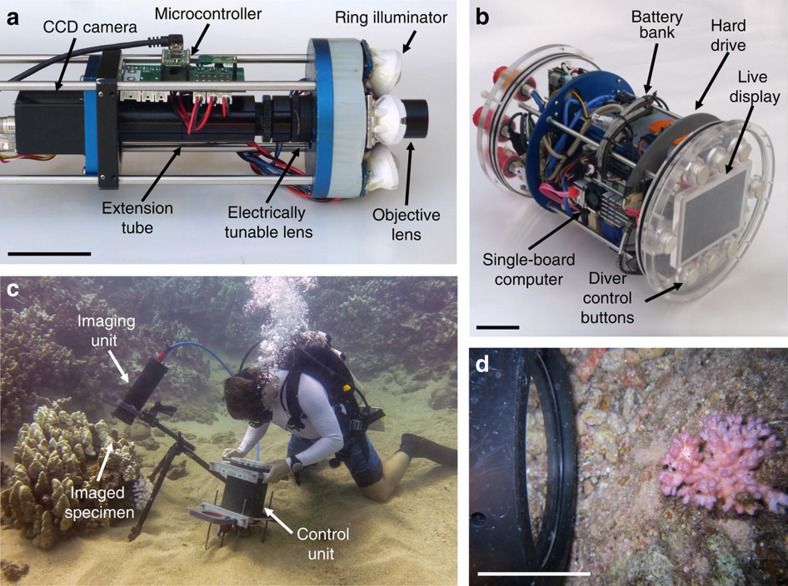
BUM design and operation. (**a**) Imaging unit internal components including illumination ring, optical lenses, CCD camera and electronic circuit board with micro-controller. (**b**) Control unit internal components including user interface with live LCD display and buttons for diver control, single-board computer and battery bank. (**c**) *In situ* underwater operation of the BUM by a scientific diver. (**d**) Close up of the BUM imaging a coral colony *in situ*, the BUM maintains a distance >65 mm from the specimen during imaging. (**a**,**b**,**d**) Scale bars, 50 mm.

Attaining non-invasive, micro-scale images in an underwater environment presents several challenges. First, microscopic imaging requires a high numerical aperture; this results in a shallow depth of field that necessitates precise focusing. Second, non-invasive imaging requires a long working distance and considerations must be made for imaging live organisms with three-dimensional structure. Finally, to perform such work underwater, rapid focusing and exposures must be used due to the unstable environment. The BUM overcomes all of these challenges through the application of three principle optical components: a long working distance microscope objective lens, a shape-changing Electrically Tunable Lens (ETL) and focused Light Emitting Diodes (LEDs) providing reflectance illumination (Fig. 1a). These elements are integrated into a compact, diver-deployed imaging system that includes a camera, electronics and user interface. The instrument is divided into two housings: the imaging unit containing all optical components, and the control unit containing a computer and a live diver interface (Fig. 1a,b).

A long working distance microscope objective lens provides the magnification and numerical aperture required to resolve fine details, as well as the working distance necessary to image through an optical port while leaving the subject undisturbed. The BUM was equipped with either a × 3 or × 5 magnification objective. As measured using a resolution target in a testing tank, the × 5 lens attained an underwater resolution of 2.2 μm with a 1.62 × 1.36 mm FOV, while the × 3 lens attained a resolution of 3.1 μm with a 2.65 × 2.22 mm FOV (Supplementary Fig. 1). Each objective provides a working distance of >65 mm from the optical port, allowing instrument set-up and imaging with minimal disturbance to the subject and its surrounding environment.

**Figure 2:**
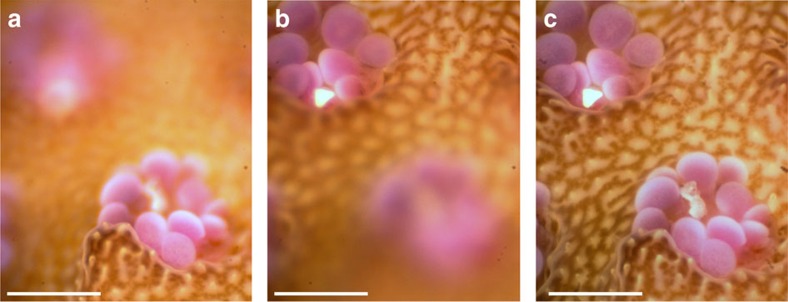
Focal scan using ETL and composite image formation. Images of a live coral acquired *in situ* with the BUM using an ETL focal scan. Images collected using the × 5 objective and wide spectrum white LED illumination. (**a**) Image of a single focal plane showing only the front coral polyp in focus. (**b**) Image of a single focal plane showing only the back coral polyp in focus. (**c**) A composite focus stacked image formed using the in-focus portions of 20 images collected with the ETL focal scan. Within a single frame (**a**,**b**), the microscope objective yields a shallow DOF. However, the composite focus stacked image, as shown in **c**, combines frames to provide an enhanced DOF such that both polyps and surrounding area are all in focus. Scale bars, 500 μm.

To achieve rapid focusing, a deformable ETL was incorporated into the optical system. This lens consists of a flexible polymer membrane encasing an optical fluid. An integrated actuator exerts variable pressure on the encased optical fluid to rapidly change the lens curvature and focal length with adjustment times of <2.5 ms (refs 21, 22). The ETL provides a compact means to bring a subject of interest into precise focus, which is a principle challenge in benthic underwater microscopy. The ETL can also rapidly scan the optical system's focal plane through a volume to bring various parts of a subject in focus. Frames can then be combined using image-processing techniques to produce a single image with all parts of the subject in focus^23^. This is commonly known as focus stacking and is an important capability for collecting observations of natural, undisturbed subjects, which often have substantial three-dimensional structure (Fig. 2). While the × 3 and × 5 objectives have narrow depths of field (34 μm and 16 μm, respectively) the ETL enables focusing over scanning ranges of 18.4 mm and 6.9 mm, respectively.

**Figure 3:**
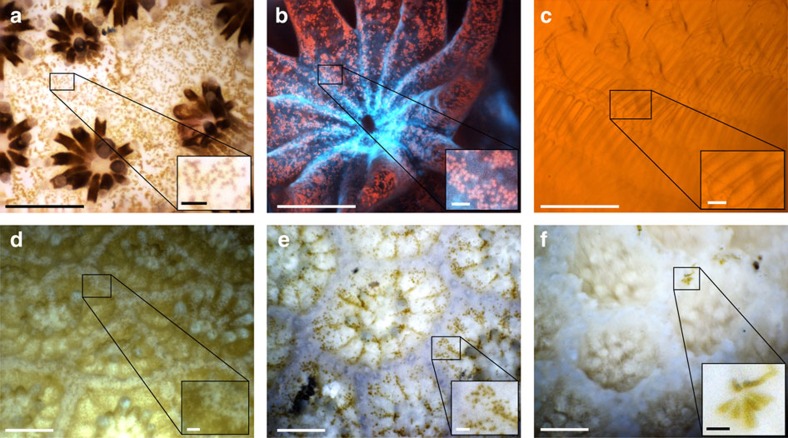
Images captured by the BUM. (**a**) *In situ* image of the coral *Stylophora* taken using the × 5 objective lens and white illumination, in Eilat, Israel. The image is an enhanced DOF composite formed from a focus stack. Individual zooxanthellae (∼6–13 μm in size) are visible in the inset. (**b**) Fluorescent image of the coral *Pocillopora* taken in a lab tank using the × 5 objective. Inset shows individual zooxanthellae emitting red fluorescence from their chlorophyll. Image is a composite focus stack. (**c**) *In situ* image of the pharyngeal basket of the semi-transparent ascidian *Rhopalaea idoneta*, taken using the × 5 objective and white illumination, in Eilat, Israel. *R. idoneta* is a filter feeder that uses the mesh of the pharyngeal basket to capture plankton. (The orange colour here is likely due to a subject behind the ascidian). (**d**,**e**) *In situ* images of two different locations on a bleaching colony of *Porites compressa*, taken in Maui, Hawaii. Images show partial bleaching in **d** and nearly complete bleaching in **e**. Images are composite focus stacks collected using the × 3 objective lens. (**f**) *In situ* image of a fully bleached colony of *Porites lobata.* Taken in Maui, Hawaii with the × 3 lens. No visible zooxanthellae can be seen in the coral; as a result the polyps have a translucent appearance. While translucent, the polyp structure and tentacles remain intact and visible, indicating that the polyp is still alive. Coenosarc tissue normally connecting polyps is either very thin, or may have fully retracted towards the polyps' centres exposing the coral skeleton. Inset shows colonization of the area between two live polyps by benthic diatoms. Main figure scale bars, 500 μm. Inset scale bars, 50 μm.

A custom designed ring of six LEDs provides the high-intensity light required for short duration, reflectance illumination. Each LED is focused with a condenser lens and angled such that all light sources converge at the plane being imaged (Fig. 1, Supplementary Fig. 2). Using illumination pulses, images were captured in the ocean with exposure times of <1 ms, which is critical for eliminating motion blur. In addition, short LED pulses reduce the total length of time organisms are exposed to artificial illumination. While we did not observe any distinct behavioural changes due to the instrument's illumination, if needed imaging can be performed at very low frequencies to further reduce artificial light effects. During most operations, wide spectrum white LEDs were used. However, fluorescence imaging was also conducted using blue LEDs combined with a long-pass optical filter in the imaging system (Fig. 3b), additionally near-infrared (NIR) LEDs were tested (Supplementary Fig. 3).

The entire system is housed in a self-contained, submersible package, which enables underwater operation of the instrument in the ocean by a single scientific diver (Fig. 1c). The instrument's physical design consists of two cylindrical aluminium housings. The imaging unit contains all optical elements, as well as a CCD camera, micro-controller and custom circuit board. The camera's imaging rate can be adjusted in a continuous range from a maximum of 15 frames per second (for rapid focal scans) to once every several minutes (for time-series recordings and reduced artificial light effects). The control unit contains an on-board computer, 500 Gb hard drive for data storage and real-time diver-controlled user interface. The BUM has a battery capacity of ∼8 h, enabling video and extended time-series recordings (during which the instrument can be left on the reef to image autonomously). In the videos shown here, the imaging unit was mounted on a tripod or rested directly on the seafloor. The tripod minimizes camera movement, facilitates non-invasive observations and enables time-series recordings. Alternatively, the instrument can be used in a hand-held mode. In this case a mechanical ranging probe (Supplementary Fig. 4) may be mounted to the imaging unit to provide a physical means for estimating the correct imaging distance, the ETL can then be scanned to image a large depth.

### Microscope imaging performance

The BUM operates while submerged, recording images with an optical resolution of up to 2.2 μm. This enables observation of fine anatomical details of organisms in both the lab and field. For example, images of live coral polyps in the ocean reveal the distribution and discrimination of individual symbiotic single-celled dinoflagellates, commonly known as zooxanthellae, living inside the coral (∼6–13 μm diameter)^24^ (Fig. 3a). With this capacity, we examined corals experiencing varying levels of bleaching (expulsion of zooxanthellae) and checked for the presence of remaining symbionts (Fig. 3d–f). Fluorescence imaging was also conducted (Fig. 3b) to further enhance observations. Corals fluoresce due to chlorophyll in their symbionts and fluorescent proteins in the animal itself, both of which inform physiological status and health^25^,^26^. Observations of other subjects, such as the plankton-trapping mesh of the ascidian *Rhopalaea idoneta* (Fig. 3c), offer opportunities to examine filter-feeding rates and biomechanics of delicate organisms. The non-invasive nature of the system allows recordings of small, fragile specimens such as the initial stages of micro algae colonizing bleached corals (Fig. 3f). Finally, *in situ* focus stacks can successfully image complex three-dimensional subjects (Figs 2 and 3a,d,e).

### Time-series and video observations

**Figure 4:**
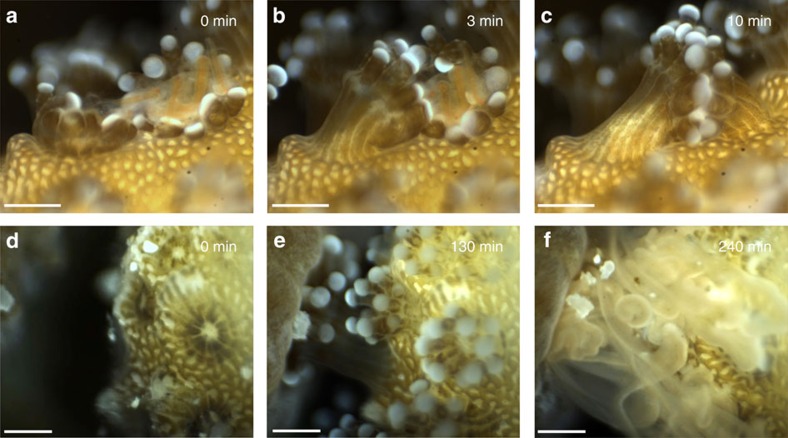
Videos captured by the BUM. (**a**–**c**) Lab images of the coral *Stylophora* showing coordinated behaviour and communal feeding between two adjacent polyps after several *Artemia*, which were injected into the tank, had been captured by the polyp on the right. Image taken using the × 3 objective, more details in Supplementary Movie 1. (**d**–**f**) *In situ* images of competition between the corals *Platygyra* (left side of images) and *Stylophora* (right side of images) collected using the × 3 objective. The *Platygyra* has emitted its mesenterial filaments and is beginning to digest the *Stylophora* in **f**. More details shown in Supplementary Movie 4. Figure scale bars, 500 μm.

Temporal observations collected by the BUM record ongoing micro-scale processes and may reveal novel phenomena. This capability is demonstrated through recordings of coral polyp behaviour, feeding and competition. During feeding studies conducted in the lab, high numbers of *Artemia* nauplii were introduced to *Stylophora* corals. Subsequent coordinated polyp behaviour was recorded in which coral polyps that had captured a high quantity of nauplii joined with neighbouring polyps by intertwining their tentacles to digest their prey (Fig. 4a–c, Supplementary Movie 1). Time-series microscopy recordings were then collected *in situ* for periods of up to 8 h to investigate the presence of these and potentially other periodic feeding activities under natural conditions. For these investigations, the BUM set-up to image autonomously on the reef overnight in the Gulf of Eilat at depths between 4 and 8 m. Here we recorded a previously undescribed behaviour, in which polyps periodically connected their gastrovascular openings throughout the night, likely exchanging materials (Supplementary Movie 2). We referred to this activity as coral ‘polyp kissing', and it often occurred after what may have been plankton capture events.

The utility of *in situ* time-series microscopy was further demonstrated through an observational study on coral–coral competition. Work was conducted on the reef in Eilat at depths between 4 and 8 m. Here loose coral colonies were moved in close proximity (∼1 mm) to one another to stimulate inter-colony competition. The BUM was fitted with the × 3 or × 5 objective lens and images of the interaction zones were autonomously recorded over the course of the night at a frame rate of 1 Hz. Pairings of different coral genera included *Stylophora* with *Pocillopora* (Supplementary Movie 3, set-up shown in Supplementary Fig. 5) and *Platygyra* with *Stylophora* (Fig. 4d–f, Supplementary Movie 4). Videos allow elucidation of competition response times, attack mechanisms such as emission of mesenterial filaments and competitive dominance between different taxa (Fig. 4d–f, Supplementary Movies 3–5). Further manipulations and a control were also staged to examine responses to differing stimuli. The coral genus *Platygyra* was paired with four different subjects: the coral *Stylophora*, the coral *Galaxea*, a conspecific *Platygyra* colony and a mesh net filled with *Artemia* (Supplementary Movie 5, Supplementary Fig. 6). Time-series videos revealed different behaviours of individual polyps when paired with different species or with conspecifics. For example, *Platygyra* quickly emitted its mesenterial filaments when paired with the coral *Galaxea*, while alternatively no aggressive behaviour or even contact was observed when it was paired with a conspecific. Observations in the natural environment of such behaviours at these spatial and temporal scales are not possible with any other available imaging system.

### Spatial pattern analysis

In addition to performing temporal recordings, the instrument can also collect images for high-resolution spatial analysis. The analysis of spatial structure is a fundamental tool used to infer underlying processes in disciplines such as ecology and physiology^10^,^27^,^28^,^29^,^30^,^31^,^32^. However, observations must be collected at scales relevant to the subject (organism or function) of interest. Via its high resolution, the BUM offers a means to collect such spatial information at new scales. Here we demonstrate this capability by examining the micro-scale spatial patterns of algae colonizing corals undergoing bleaching.

During the summer and fall of 2015, the Main Hawaiian Islands experienced their first recorded mass coral bleaching event. Bleached corals lost their symbiotic zooxanthellae, but remained alive, with polyps still visible (Fig. 3d–f). However, following bleaching, many corals on the island of Maui experienced rapid overgrowth by communities of benthic diatoms and turf algal filaments (Supplementary Fig. 7). This overgrowth was associated with the decline and ultimate death of these colonies (Supplementary Fig. 8). The algal overgrowth of bleached corals while still alive has rarely been documented at such significant magnitudes. Relatively little is known regarding the specific patterns and mechanism by which these algae are rapidly colonizing and overgrowing living corals^33^,^34^. In addition, lab techniques for investigating such small-scale interactions would require destructive sampling to retrieve specimens.

**Figure 5:**
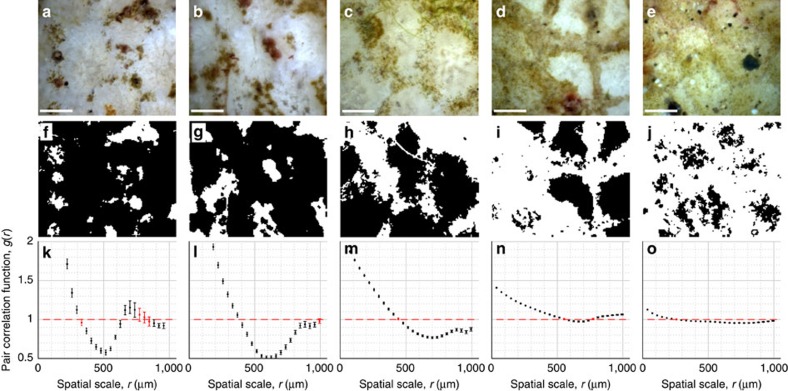
Spatial analysis of algal overgrowth on bleached coral. (**a**–**e**) *In situ* images acquired at the Kahekili reef site in West Maui showing different successional stages of a filamentous algal overgrowth on bleached *Porites lobata*. All images were captured on the same dive. The × 3 objective lens was used providing a 2.82 × 2.36 mm field-of-view. The structure of live coral polyps can be seen on close examination in **a**,**c**, polyps are dead in **e** and images are inconclusive for **b**,**d**. Algae are on surface (not endolithic) as they were observed swaying in the water over several images. (**f**–**j**) Categorical raster maps showing locations of algae represented by white cells. The raster map consists of 153 × 128 grid cells (each 18.4 × 18.4 μm^2^ in size) each representing the presence or absence of algae. Algae segmentation was performed using a global saturation threshold. (**k**–**o**) Pair-correlation function, *g*(*r*), values calculated using the algal raster map (details in Methods); *g*(*r*)>1 indicates clustering, and *g*(*r*)<1 indicates regularity, where radius *r* is the spatial scale being considered. The *g*(*r*) values include 95% confidence intervals, which were calculated using a method of resampling without replacement^30^. Each confidence interval was determined from the distribution of *g*(*r*) values calculated from 999 resampling draws of half the population (see Methods). *g*(*r*) points are red if the confidence interval overlaps with *g*(*r*)=1 (which is consistent with complete spatial randomness (CSR)), otherwise points are black indicating statistically significant deviation from CSR. The strong patterns in *g*(*r*) indicate spatial order. At small scales, algae are strongly clustered, there is then a distinct dip and recovery in *g*(*r*), indicating a region with reduced algal density, likely due to exclusion by coral polyps. Figure scale bars, 500 μm.

To more closely examine this process in its natural, undisturbed state the BUM was used *in situ* to image communities of benthic diatoms and turf algal filaments growing on bleached *Porites lobata.* Varying successional states of algal colonization were observed by imaging different locations on the corals' surfaces (Fig. 5a–e). *In situ* images show algae colonizing the surfaces of living corals in which polyps were clearly visible (Figs 3f,5a,c; Supplementary Fig. 9). Images were used to investigate whether algal colonization of bleached corals was occurring in a random or non-random pattern. First image-processing techniques were applied to segment algae, then algal spatial distributions were analysed using *g*(*r*), the pair-correlation function (PCF) (Fig. 5) (see Methods)^28^,^29^,^30^,^31^,^32^. The *g*(*r*) value is a measure of the relative density of neighbouring conspecifics (similar individuals) around an average individual as a function of radius, *r*. This metric allows analysis of clustering at multiple independent length scales. If more neighbours are present at a given radius than would be expected with a random arrangement then, *g*(*r*)>1, indicating clustering (aggregation). Alternatively if fewer subjects are present than expected then *g*(*r*)<1, indicating regularity (overdispersion) (details in Methods).

The PCF analyses show strong patterning in *g*(*r*), indicating high levels of spatial order that demonstrates the algae are not randomly arranged^29^,^30^. Specifically, the PCF plots indicate three critical scales of interest (Fig. 5k–o). At small scales, *r* less than ∼300 μm, high *g*(*r*) values denote significant aggregation. At intermediate scales, *r* in the range of 500–700 μm, *g*(*r*) values reach a distinct minimum around which there is a relatively symmetrical dip. Finally at larger scales, *r* greater than ∼700–900 μm, *g*(*r*) returns to values much closer to randomness (*g*(*r*)=1). In summary, the PCF reveals a mixed pattern: algae are clustered with high densities at small scales, experience a zone of relative exclusion with low densities at intermediate scales and then return to moderate densities at larger scales. This structure suggests a pattern formed by interactions between the algae and coral. Strong small-scale aggregation indicates that algal communities grow in patches. The subsequent dip and distinct minimum in *g*(*r*) reflects the existence of an exclusionary zone, occurring at a regular interval, in which algal densities are significantly lower than expected under randomness^29^. The distance to this *g*(*r*) minimum is slightly larger than the radius of a *Porites* polyp, and is likely indicative of algae clustering at the edge of a polyp with their presence being inhibited close to the polyp centre. Finally, the increase in *g*(*r*) at larger radii suggests that algae patches occur separated by gaps, for example, on either side of a polyp. Comparing PCF plots, the general three-phase pattern is observed in Fig. 5a–d; however, the intensity of *g*(*r*) patterning decreases with increasing algal density. The results of this preliminary *in situ* microscopy investigation show that algae can colonize living bleached corals and suggests that they may do so by establishing clusters on the ridges between adjacent coral polyps, resulting in the honeycomb pattern observed in Fig. 5. Further, these images demonstrate the BUM's ability to collect ecologically significant spatial data that can be used for quantitative analysis.

## Discussion

Using the novel BUM, we have demonstrated the first *in situ* imaging of benthic marine organisms at nearly micrometre resolution. The methods described here provide the means for rapid exposures, active focusing and a long working distance. This facilitates non-invasive microscopy of live three-dimensional subjects in the ocean. Several scientific applications of the system were demonstrated through field observations on coral reefs. The instrument enabled time-series recordings of new organism behaviour, as well as observations of important biological patterns at heretofore unobserved scales.

*In situ* microscopy videos revealed novel coral behaviours, where individual polyps periodically connected their gastrovascular openings in what is likely to be a mechanism for resource sharing. While previous studies have demonstrated transfer of energetic products via connective tissue between polyps^35^ additional resource sharing mechanisms have been hypothesized^36^ but not directly observed. In addition, we document detailed interactions within and between coral species, including the competitive dominance of certain taxa. These interactions could be further studied with more replicated observations. The recordings show that corals have the capacity to differentiate between conspecifics and other competitors, highlighting several interesting hypotheses (such as chemical, microbial interactions) that could be tested using the BUM with controlled *in situ* experiments^37^,^38^.

The system's ability to collect meaningful spatial data was demonstrated through *in situ* microscopy of bleached corals that were being colonized by filamentous turf algae. To characterize the algae's micro-scale spatial patterns, the images were analysed using a second order spatial statistics approach (PCF). The analysis supports the hypothesis that algae are distributed in significant patterns, and that these patterns can be statistically observed within single microscope images. Further, the initial stages of succession appear to be characterized by algae clustering in patches between coral polyps. Although more complete studies with repetition would be necessary to completely validate this interpretation, these initial observations merit some mechanistic consideration. First, the exclusion of algae from large gaps indicates that, as we interpret it, little or no algae may settle directly on live polyps^34^. The clustering between polyps also suggests that the thin coenosarc tissue connecting polyps may be weakened or retract during bleaching (such as observed before polyp bailout^39^), exposing the underlying skeleton and providing algae a critical surface on which to initially settle^40^. However, it is important to note that alternate processes may also generate the observed patterns so further testing is needed to confirm any specific mechanisms. Finally, algae distributions may exhibit systematic changes with increasing density; such as increasing algal patch connectivity, resulting in the isolation of individual polyps. Further sampling and analysis offer a means to reach quantitative conclusions regarding changes in algal spatial distributions as function of density. Here we examined one important case of coral–algal interaction, in alternate cases patterns of algal colonization and succession may vary considerably based on the species of coral and algae involved, as well as the conditions they are exposed to ref. 33. However, most importantly for the discussion here, results demonstrate that the BUM can record images that can be processed to obtain quantitative *in situ* spatial data for testing specific hypotheses.

Natural processes occurring in benthic marine environments span extensive spatial and temporal scales (Supplementary Fig. 10), and in many cases small-scale processes drive the structure and health of the larger ecosystem^10^. For example, while corals create massive reefs that can be viewed from satellites, individual coral polyps are typically on the order of a millimetre in size. In terrestrial ecology, the principle of the ‘plant's eye view' of a community has been used to emphasize the importance of studying subjects at scales relevant to individual organisms and the local environments they directly experience^32^,^41^; here we enable that perspective for marine subjects, including a ‘coral polyp's eye view'. Previous observations of micro-scale benthic marine processes have been confined to the lab, imposing significant limitations on what can be observed. Underwater microscopy thus offers a new means to study behaviours and interactions in the natural environment that may otherwise remain unresolved or poorly understood. This is of heightened importance as coral reefs around the world are facing increasing stress from anthropogenic activities, resulting in declines in coral cover on many reefs^3^,^19^,^20^. However in many cases, the mechanisms and details of these declines are not fully understood. The BUM has potential to provide new insights on diverse ecological topics directly relevant to these declines such as competitive dynamics at the thin algal–coral interfaces^3^,^4^,^5^, coral bleaching and recovery^1^,^42^, disease dynamics^43^,^44^, and larval settlement and growth. As a versatile tool, underwater microscopy has clear and viable applications in a wide variety of scientific fields including ecology, physiology, biomechanics^2^,^45^, fluid dynamics, marine geology and physical-biological coupling^7^,^46^.

Finally, the instrument offers a platform for technology development. A variety of enhanced imaging methods may be incorporated into future designs to provide additional information on micro-scale physical and biological phenomena. Such techniques include variable chlorophyll fluorescence imaging to study photosynthetic efficiency^25^; micro-particle image velocimetry to study small-scale fluid dynamics^47^,^48^; chemical measurements using optical indicators^25^,^49^; and three-dimensional modelling of imaged subjects using the depth information that is available from the ETL focus stacks. We hope that insights from the current instrument design and deployment methods will provide a foundation for both increasingly powerful *in situ* imaging, and a wave of bringing lab research into the ocean. This *in situ* viewpoint enables novel investigations on basic marine research and will provide the means to connect theoretical lab work to the natural environment.

## Methods

### Optical system

The optical system is modular and accommodates standard C-mount long working distance objective lenses. Here we use two different telecentric finite conjugate objectives (Mitutoyo ML × 5, ML × 3). Light entering the microscope passes from the water through an acrylic optical port (5.6-mm-thick) and into the objective lens. The numerical aperture (NA=*n* × sin(*θ*), where *n* is the index of refraction and *θ* is the half angle of the light cone entering the lens) of the optical system is conserved across the port's flat-refracting boundaries, following Snell's Law. However, the instrument's working distance increases due to water's higher index of refraction.

The shape-changing ETL (Optotune EL-10-30-C) provides fast electrically controlled changes in focal length. The ETL has a back focal length range of 20–200 mm. It can be paired with a −150 mm meniscus offset lens such that the combined lenses have a focal length range of −130–50 mm. The optimal position for the ETL is the back focal plane of the microscope objective. However this plane was inaccessible, so the ETL was positioned just after the objective lens. As a result, the size of the FOV changes by ∼13% in both width and height over the focal scan. The FOV can be adjusted in post processing for focus stacks so that all images are the same size.

Images are recorded by a machine vision Prosilica GC2450 colour CCD camera (12 bit dynamic range, 2,448 × 2,050 pixels, 3.45 μm pixel pitch, maximum frame rate of 15 fps at full resolution, global shutter). The ring illuminator (Supplementary Fig. 2) contains six LEDs with emission wavelengths for either white light reflectance illumination (LED Engin LZ1-00WW00, warm-white), NIR reflectance illumination (LED Engin LZ1, far-red peak at 740 nm) or fluorescence (LED Engin LZ1-00DB00, peak at 460 nm) imaging. Each LED is focused using an aspheric condenser lens (27 mm Diameter × 13 mm FL, Edmund Optics 43–987). The illumination ring and condenser lens mounts were fabricated using three-dimensional printing. During fluorescence imaging band pass excitation filters, transmitting between 419 and 465 nm (SemRock, CFW-BP01), are mounted on the LEDs and a long-pass emission filter, cutoff of 473 nm (SemRock, BLP01-473R), is mounted behind the objective lens.

### Housings and electronics

The housings for the control unit (30.6 cm length × 21.9 cm diameter) and imaging unit (39.5 cm length × 12.2 cm diameter) were constructed from 6061 aluminium cylinders. End caps were machined from optically clear sheets of Spartech Polycast Super Abrasion Resistant acrylic (5.6-mm-thick for the optical housing and 25-mm-thick for the control housing). The housings were pressure tested to a depth of 30 m, sufficient for normal SCUBA operations. In addition pressure ratings of up to 300 m can be achieved by using thicker end caps. A diving frame machined from PVC sheets holds both housings during underwater deployments. The entire system weighs ∼23 kg in air, but is only slightly negatively buoyant underwater.

The control unit houses a Pico-ITX embedded motherboard (LP-170, Global American), 500 Gb solid-state hard drive and four Li-Ion batteries (Ocean Server) permitting ∼8 h of system operation. Real-time, underwater user control is achieved through a 5 inch LCD screen (Purdy Electronics), and eight piezo-electric buttons (Baran Advanced Technologies). A graphical user interface enables live viewing of acquired images and full instrument operational control; software was written in C++ and based on the camera's SDK. The control and imaging units are connected via a 2 m underwater cable (SubConn) supporting Ethernet, USB and power connections.

The opto-electronics in the imaging unit are controlled by a micro-controller (Teensy 2.0, Arduino code) mounted on a custom PCB. This system synchronizes the camera shutter, illumination LEDs and the ETL. The ETL's focal length changes as a function of a supplied current, which is adjusted by a precision current controller (ADN8810) mounted on the PCB. The PCB also supports two LED illumination modes: a high current supplied during imaging for pulsed illumination, and a low current supplied for low intensity continuous illumination. The control unit's motherboard is connected to the imaging unit's micro-controller via USB and the CCD camera via Ethernet.

### Image processing

Image focus stacks were combined to form enhanced DOF images using a commercial software package (Helicon Focus). For display, linear image contrast stretching and brightening was applied. In addition, specular reflections in resolution target images were eliminated by applying a threshold to reduce anomalously bright spots.

The videos were processed using an open source software package (Virtual Dub). This package was used to stabilize Supplementary Movie 3; additionally small smudges on the camera sensor were removed from Supplementary Movies 1, 3 and 4. Finally, linear image contrast stretching and brightening were applied. Videos were compressed using the open source FFmpeg software package.

### Lab measurement of optical performance

The instrument's resolving power was quantified in the lab using a 1951 USAF resolution target mounted on a white PVC sheet. The resolution target was designed for transmission illumination (we were unable to find a target designed for reflectance imaging with small enough resolution bars), and as a result target images may show reduced contrast compared with other specimen. Imaging performance tests were conducted in a test tank filled with seawater, as well as in air. The resolution target was positioned using a micromanipulator with a movement accuracy of 0.025 mm. Maximum resolution in seawater was measured to be 2.19 μm with the × 5 lens and 3.11 μm with the × 3 lens (Supplementary Fig. 1).

It was also necessary to consider two sources of changing aberrations in the system caused by the ETL. First, the flexible membrane of the ETL lens sags slightly due to gravity, creating asymmetry in the lens. Thus for optimal imaging, the ETL should be placed in a vertical position, with the optical axis parallel to the gravity. In the horizontal position the BUM's underwater resolution decreased to 2.46 μm with the × 5 lens and 3.91 μm with the × 3 lens. Second, objective lenses are designed for operation at a specific working distance. However, as the ETL focal distance changes it moves the objective lens away from its designed working distance. This results in reduced image resolution and contrast. Resolution at the largest ETL deformation was measured to be 3.47 μm and 4.39 μm for the × 5 and × 3 lenses, respectively, largely due to decreased image contrast. Finally, the system's FOV was measured using a Ronchi ruling with five lines per millimetre, and working distance and scan range were measured using the micromanipulator to control the target's location. Values are reported in Supplementary Table 1.

### Underwater operation

The BUM is designed for deployment and operation in the ocean by a scientific diver. To facilitate optimal functionality the instrument is self-contained, slightly negatively buoyant underwater, and split into two modules. The imaging unit is smaller to enable easy maneuvering and positioning. It is tethered to a larger control housing, which is separate to avoid inadvertent movement of the system's optics during user control. Challenges during the diver operation include positioning the imaging unit at the correct working distance and adjusting the desired field-of-view. With this in mind, the instrument provides an LCD display with live feedback. In addition, the six LEDs in the ring illuminator can provide continuous low intensity illumination. All six light sources are focused and converge at the point being imaged (Supplementary Fig. 2). This provides a visual aid for ranging and framing the subject of interest. Finally, fine focusing is conducted by controlling the ETL.

During time-lapse recordings and continuous imaging the imaging unit was mounted on a portable tripod (Vanguard Alta Pro 263AGH), which stabilized the instrument and provided several degrees of freedom (including a critical ball mount pistol grip). The tripod was not designed for underwater applications and eventually failed after ∼25 dives due to saltwater exposure, future efforts will likely include the fabrication of a similar tripod using saltwater-resistant materials. Using this set-up, the organism is never physically disturbed and divers were able to complete tripod and instrument positioning within ∼5 min, after which many images of a site can be acquired. In addition, the system was left to perform overnight imaging on the reef autonomously. Alternatively, the imaging unit can be used in a hand-held mode. In this case, a mechanical ranging probe (Supplementary Fig. 3) can be mounted around the imaging port. The ranging probe comes in contact with the substrate around the subject being imaged to indicate approximate working distance and aid instrument stability. A complete focus stack is then rapidly acquired using the ETL and the in-focus planes are selected later in the lab. This method is invasive and not ideal for fragile organisms such as corals, but can be useful for other subjects growing on firm substrates such as rocky reefs.

### Spatial pattern analysis

The spatial patterns of algae colonizing bleached corals were analysed using the PCF. The PCF, *g*(*r*), is a noncumulative neighbourhood density function, where *r* is the distance between two neighbours. It is determined by counting the number of neighbours surrounding each subject in annuli of constant width and increasing radius. Densities within each annulus are determined by dividing neighbour counts by the annulus area. Annulus densities for a given radius, *r*, are then averaged for all subjects. Finally, averaged densities at each radius are normalized by the average density of the entire image to obtain *g*(*r*) values^28^,^30^,^32^. This metric represents relative density as a function of distance. It is well-suited to reveal mixed patterns of aggregation and dispersion at different critical scales. It is relevant to note that while the PCF is used here, a host of spatial statistics for a variety of applications exist and may be applied to future microscopy images^27^.

To apply the PCF, we segmented algae from the rest of the microscopic image (Fig. 5f–j). First, 2 × 2 neighbourhoods of pixels were averaged to reduce noise in raw images. Then, the colour space was converted from RGB to HSV. Finally, a global saturation threshold was applied to distinguish pixels occupied by algae. Saturation indicates the degree to which a given pixel's colour channels vary in intensity. Bleached coral tissue reflects light approximately evenly, producing low pixel saturation values. Alternatively, algae exhibit preferential light absorption at specific wavelengths, producing higher pixel saturation values. An expert user manually selected a saturation threshold value of 0.25 and this value was applied consistently across all segmented images. (To demonstrate that final results were not highly sensitive to small changes in the threshold value selected, we also applied the PCF analysis to images segmented using saturation thresholds of 0.22 and 0.28. The *g*(*r*) values produced when using these thresholds differed by an average of <4%, from the *g*(*r*) values produced when using the 0.25 threshold). Image segmentation results were used to create a categorical raster grid with each grid cell indicating presence or absence of algae^28^,^32^. A cell size of 18.4 × 18.4 μm^2^ (equivalent to 16 × 16 camera pixels) was selected as this is the approximate size of the small algal filaments in the image (and thus the minimum resolution needed to capture features of interest). Each raster map consisted 153 × 128 cells. It is important to note that while a simple segmentation approach is used here, many different computational techniques may be applied in the future to examine and extract different features from microscopy images^50^.

The PCF was then applied to this raster map^32^ using the grid-based approach presented by Wiegand and Moloney^28^, with an annulus width equal to two cells. To prevent edge effects, a buffer zone technique was applied, in which annuli were only used if they were fully contained within the imaged area^28^. Each *g*(*r*) data point was attained using annulus counts around a minimum of at least 100 individual cells (this resulted in a total of at least 8,000 individual point-to-point distances being used to calculate each *g*(*r*) value). Second order spatial statistics (such as the PCF) typically operate under assumptions that the field being studied is isotropic and homogeneous (intensity of pattern does not vary over image). However, because the PCF is a scale-dependent density function, it is less sensitive to effects of non-homogeneity than cumulative second order statistic such as Ripley's *K*-function; and the pattern of *g*(*r*) itself can be used to detect non-homogeneity^28^. A *g*(*r*) value that trends to 1 for large scales indicates homogeneity^29^; this is observed in Fig. 5a,b, which also show the most distinct spatial patterns. However, in Fig. 5c,d values of *g*(*r*) deviate slightly from *g*(*r*)=1 at large scales, this indicates that these plots may be somewhat biased by non-homogeneity, as a result their patterns may be influenced by virtual aggregation^28^.

To estimate confidence intervals, a resampling approach was applied based on the method and interpretations presented by Condit *et al*.^30^, as follows here. For each radius*, r*, a random sample of half the population was repeatedly drawn without replacement and its *g*(*r*) calculated. This was repeated for a total of 999 resampling draws of half the population. A 95% confidence interval was then established by selecting the 25th lowest and 25th highest *g*(*r*) values of this sampling distribution. Clustering or regularity was inferred if confidence intervals were either entirely above or below *g*(*r*)=1, which is consistent with complete spatial randomness (a homogeneous Poisson process). Distance classes were judged as different if confidence intervals did not overlap^30^. All image processing and statistical analyses were performed using Matlab.

### Data availability

All data supporting the biological findings of this study are available within the article and its Supplementary Information Files. Original and full resolution images are available from the corresponding author on request. Data supporting the optical resolution of the system are contained within the Supplementary Images; additional calibration images are available from the corresponding author upon request.

## Additional information

**How to cite this article:** Mullen, A. D. *et al*. Underwater microscopy for *in situ* studies of benthic ecosystems. *Nat. Commun.* 7:12093 doi: 10.1038/ncomms12093 (2016).

## Supplementary Material

Supplementary Figure and Supplementary Table Supplementary Figures 1-10 and Supplementary Table 1

Supplementary Movie 1 Coral polyp communal feeding. Lab time series video of the coral *Stylophora* showing communal feeding between two adjacent polyps after injection of *Artemia* near the coral. Images were captured using the 3x objective (2.65 x 2.22 mm FOV) and a frame rate of 5 FPS. The video is played back at 48x live speed; the upper right corner shows the video's total elapsed time.

Supplementary Movie 2 Coral 'polyp kissing'. In situ time series video of the coral *Stylophora*. The video shows periodic interactions between adjacent coral polyps involving the touching of their gastrovascular cavity openings. Images were captured over a period of six hours during the night using the 3x objective (2.65 x 2.22 mm FOV) and a frame rate of 1 FPS. The video is played back at 24x live speed; the upper right corner shows the total elapsed time since video recording began.

Supplementary Movie 3 Coral completion between *Stylophora* and *Pocillopora*. In situ time series video of competition between the corals *Stylophora* (left) and *Pocillopora* (right). Both colonies were attached to loose rock on the reef and were moved in close proximity to each other in order to induce competition (setup shown in Supplementary Figure 5). Images were captured at night using the 5x objective (1.62 x 1.36 mm FOV) and a frame rate of 1 FPS. The video is played back at 120x live speed, covering approximately 5.5 hours.

Supplementary Movie 4 Coral completion between *Platygyra* and *Stylophora*. In situ time series video of competition between the corals *Platygyra* (left) and *Stylophora* (right). The *Stylophora* colony was a loose fragment found on the reef that was moved in close proximity to the *Platygyra* in order to induce competition. Images were captured at night using the 3x objective (2.65 x 2.22 mm FOV) and a frame rate of 1 FPS. The video is played back at 480x live speed, the upper right corner shows the video's total elapsed time.

Supplementary Movie 5 *Platygyra* exposed to four different stimuli. In situ time series video of the interactions between colonies of the coral *Platygyra* and four different stimuli. In each frame the *Platygyra* colony is on the left side. *Platygyra* is paired with the following stimuli: top-left - a small colony of the coral *Galaxea* brought from the lab, bottom left - a mesh net filled with *Artemia* (brine shrimp), top right - a loose colony of the coral *Stylophora* moved from nearby on the reef, and bottom right - a loose foreign colony of *Platygyra* moved from nearby on the reef. Images were captured at night using the 3x objective (2.65 x 2.22 mm FOV) and a frame rate of 1 FPS. The video is played back at 480x live speed; the upper right corner in each frame shows the videos total elapsed time. Macro images of each pairing are shown in Supplementary Fig. 6.
