# Supplementary material for: Underwater microscopy for in situ studies of benthic ecosystems
Source: Nat Commun. 2016 Jul 12;7:12093. doi: 10.1038/ncomms12093 (PMC4945872; doi:10.1038/ncomms12093)
Supplement: Supplementary Figure and Supplementary Table — Supplementary Figures 1-10 and Supplementary Table 1 [file ncomms12093-s1.pdf]

Supplementary Figures:

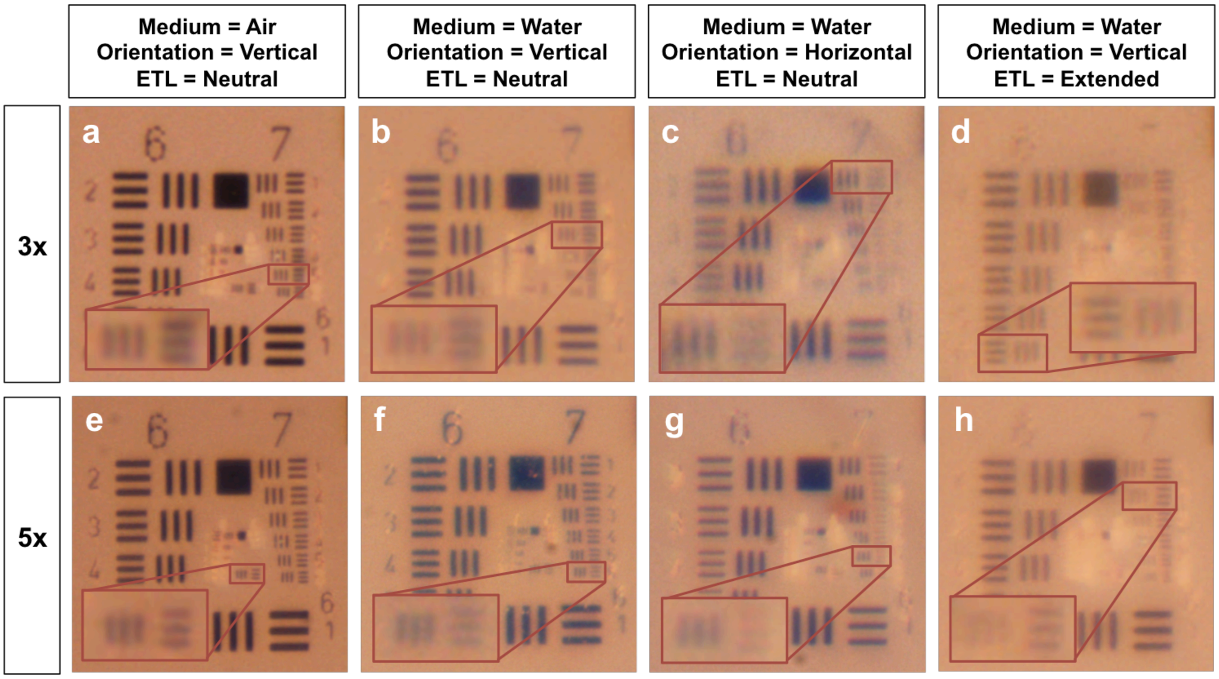

Supplementary Figure 1 | Resolution target. Images of a 1951 USAF calibration target taken with the BUM under different imaging conditions using the 3x and 5x objective lenses. Insets zoom in on the smallest resolvable bars of each test image. (**a**,**e**) Images in air with the BUM imaging unit placed in a vertical position and the ETL in a neutral setting. (**b**,**f**) Images in water with the BUM imaging unit placed in a vertical position and the ETL in a neutral setting. (**c**,**g**) Images in water with the BUM imaging unit placed in a horizontal position and the ETL in a neutral setting. (**d**,**h**) Images in water with the BUM imaging unit placed in a vertical position and the ETL in an extended setting. The highest underwater resolution for the objective lenses was attained in **b** and **f**, where the instrument was oriented vertically and the ETL set to a neutral state. Under these conditions the 3x lens was able to resolve group 7 element 3 (3.10 µm bar width) and the 5x lens was able to resolve group 7 element 6 (2.19 µm bar width).

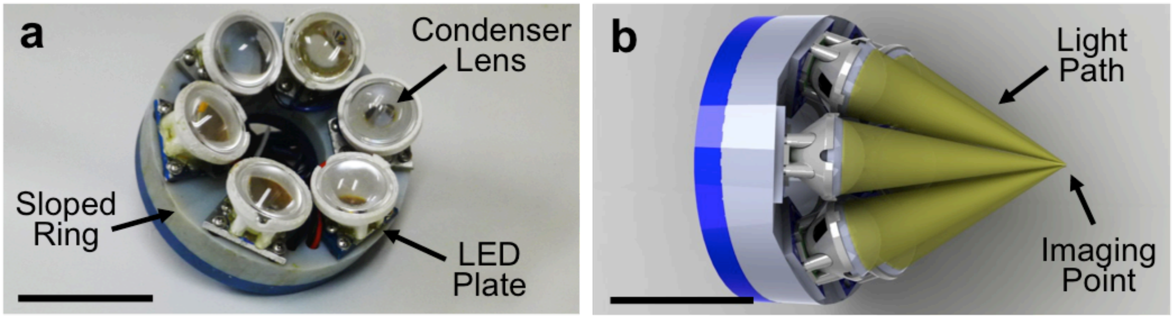

Supplementary Figure 2 | BUM illumination ring. (**a**) Image of the illumination ring, the design consists of 6 angled LEDs each focused with a condenser lens. (**b**) Digital rendering of the illumination ring, showing light paths of all LED's converge at the imaging plane.

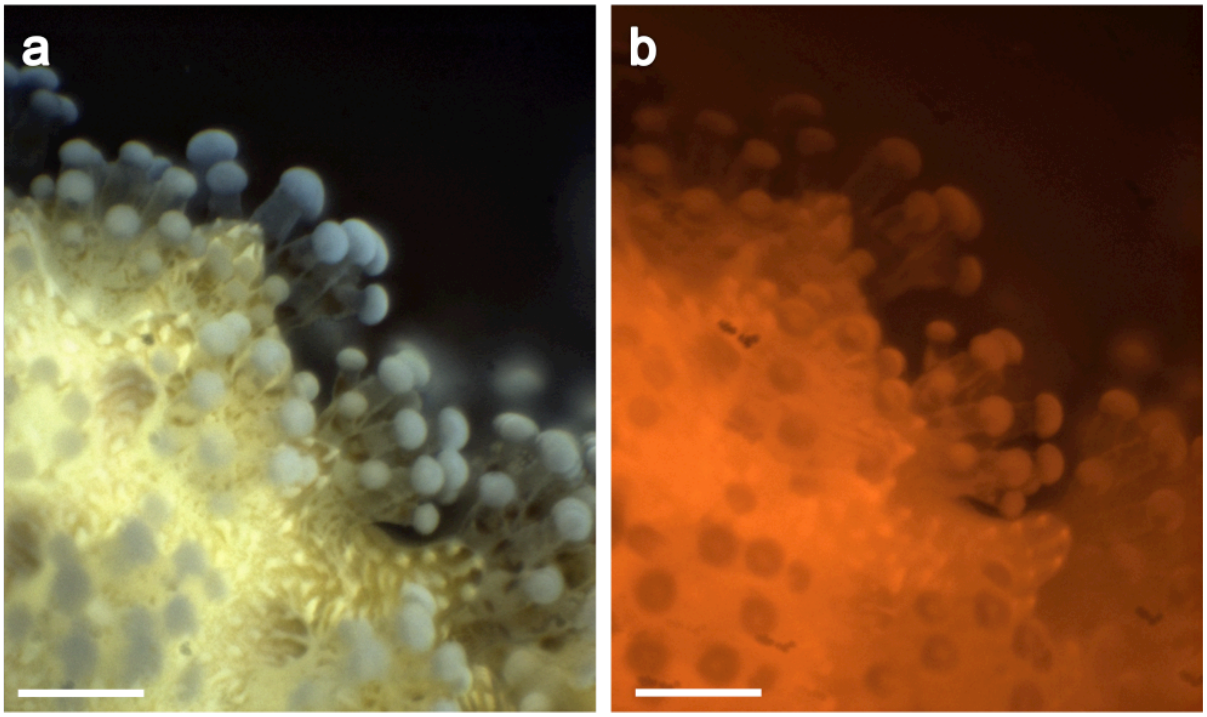

Supplementary Figure 3 | Near-IR illumination . *In situ* image of the coral *Stylophora* taken using the 3x objective lens. (**a**) Image taken using white reflectance illumination. (**b**) Image taken using near-IR reflectance illumination. Scale bars, 500 µm.

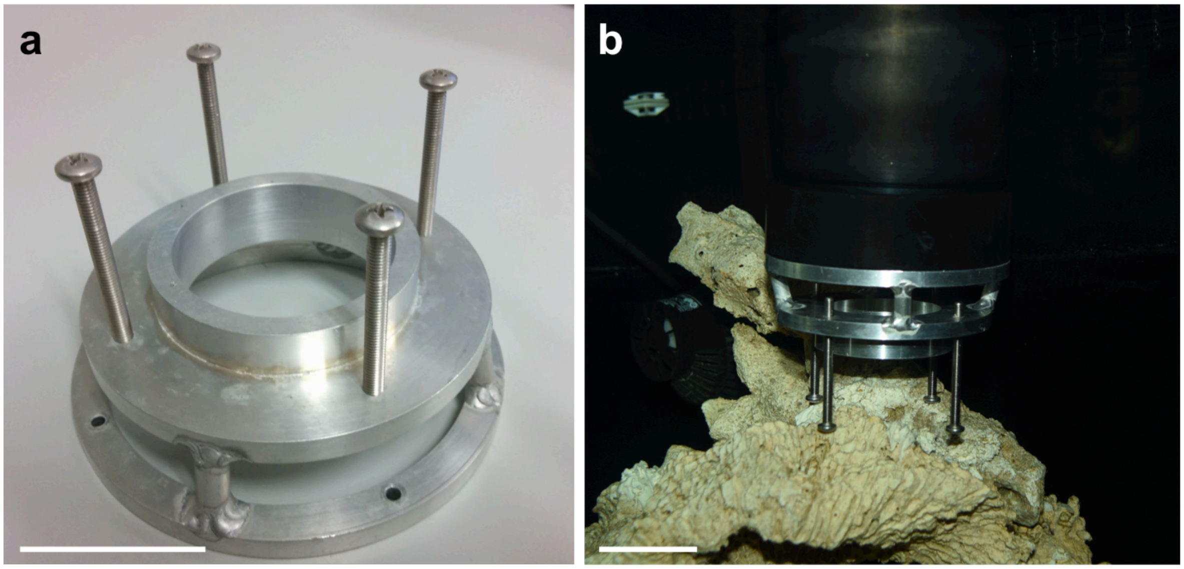

Supplementary Figure 4 | Ranging probe. A mechanical ranging probe may be mounted around the imaging port in order to facilitate handheld instrument operation. The probe can be pressed against the substrate being imaged, such as a rocky reef, in order to provide the optics housing stability and correct ranging. (**a**) Ranging probe. (**b**) Ranging probe mounted to the imaging unit, and its use being demonstrated in an aquarium. Scale bars, 50 mm.

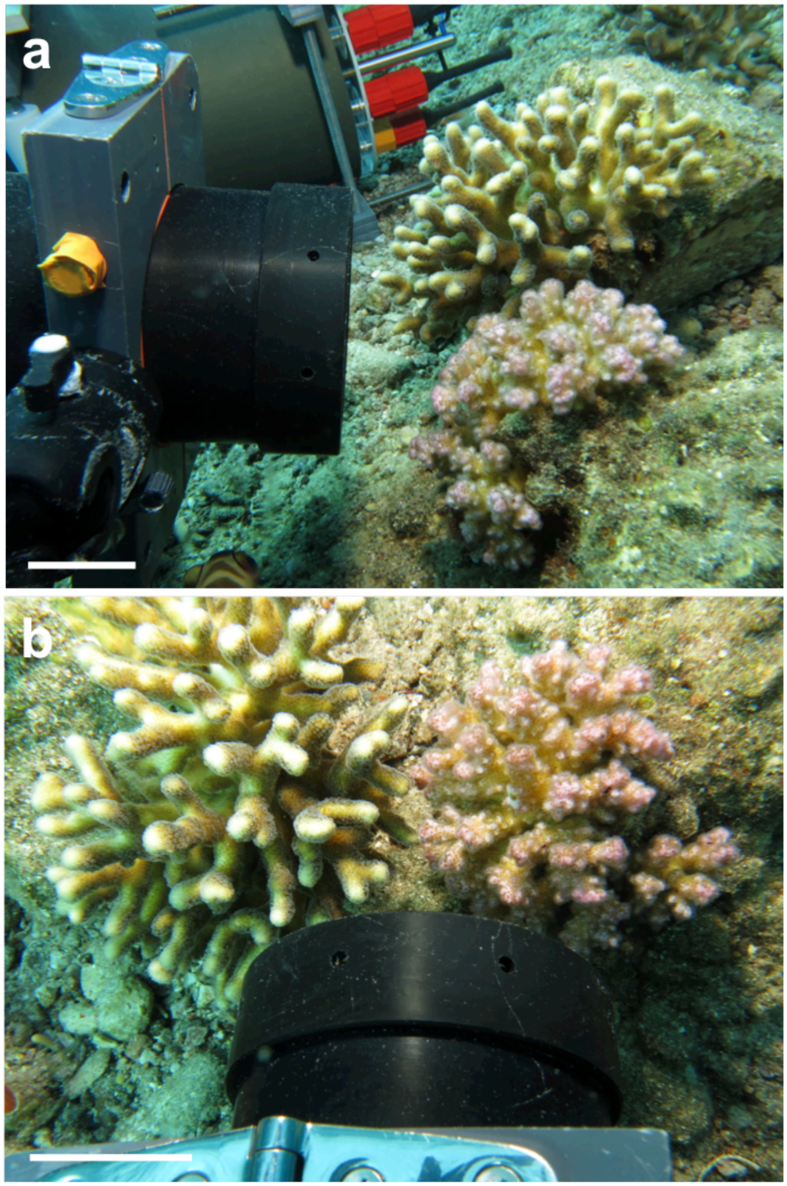

Supplementary Figure 5 | Coral competition video recording setup. (**a**-**b**) *In situ* setup of the BUM during the recording of Supplementary Movie 3. Loose coral colonies of *Stylophora* (left coral in **b**) and *Pocillopora* (right coral in **b**) were moved in close proximity (approximately 1mm) to one another. The BUM maintains a distance of greater than 65mm from the interaction area imaged. Once set in place, the BUM was left to image the coral's interaction autonomously over the course of a night. Scale bars, 50 mm.

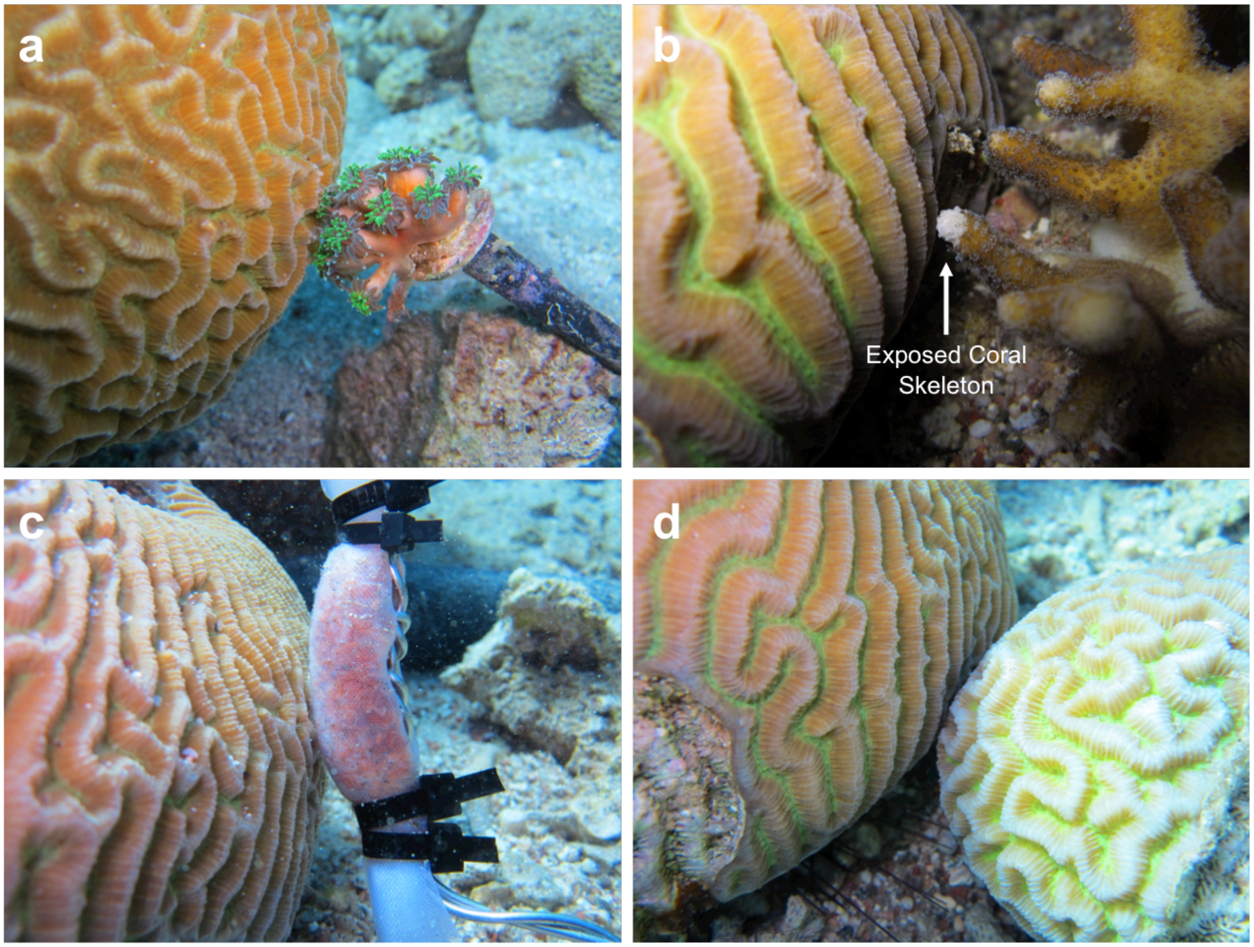

Supplementary Figure 6 | Coral *Platygyra* paired with four different stimuli. Macro images of the *in situ* organism pairings shown in Supplementary Movie 5. All images were taken after the interactions recorded in Supplementary Movie 5 had occurred. (**a**) The coral *Platygyra* paired with a small colony of the coral *Galaxea* that was brought from the lab. During the previous night the corals competed (Supplementary Movie 5). No damage to either coral is clearly visible in this image, however upon removal one of the *Galaxea* polyps was observed to have been killed. (**b**) The coral *Platygyra* paired with a loose colony of the coral *Stylophora*. The *Stylophora* colony was moved from a nearby site on the reef. The tissue on the tip of one of the *Stylophora's* branches was digested by the *Platygyra's* mesenterial filaments during the night (Supplementary Movie 5). The white skeleton of the *Stylophora* is now exposed where its tissue was digested by the *Platygyra*. (**c**) The coral *Platygyra* paired with a mesh net filled with *Artemia* (brine shrimp) prepared in the lab. During the previous night the *Platygyra* used its mesenterial filaments to digest some of the *Artemia* in the net (Supplementary Movie 5). (**d**) The coral *Platygyra* paired with a loose colony of the same genus that was moved from nearby on the reef. The two colonies of the same genus displayed no aggressive behavior or even contact during the recorded microscopy video from the previous night (Supplementary Movie 5). Based on the coral's features, images **a**, **b**, **c**, and **d** have fields of view with widths of approximately 75 mm to 150 mm.

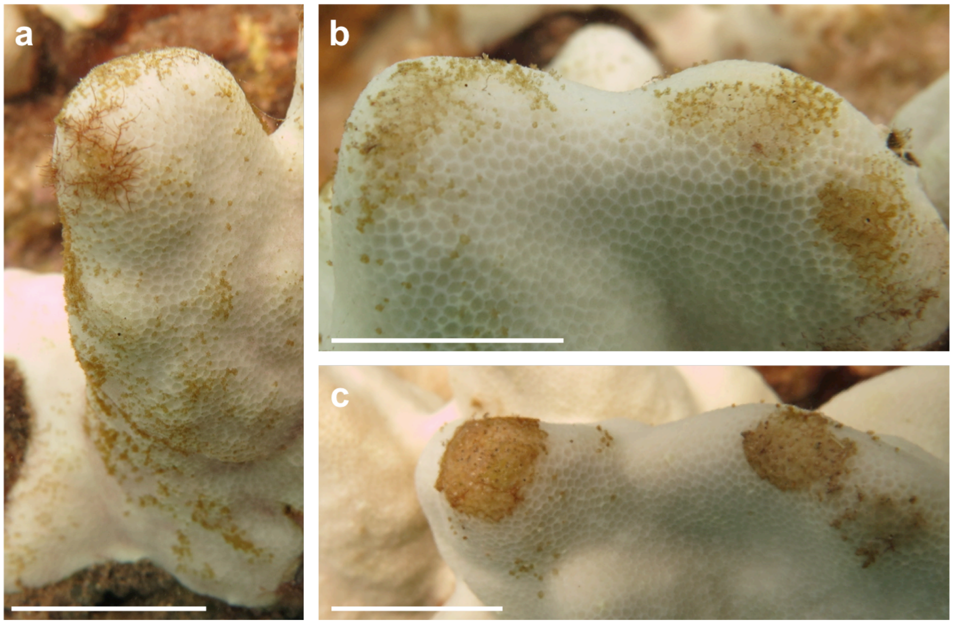

Supplementary Figure 7 | Initial algal colonization of bleached *Porites* coral colonies. (**a-c**) Images taken with a conventional underwater camera in Maui, Hawaii. At this stage the coral is bleached but the majority of polyps are most likely still alive. Algae exist primarily on the walls between individual coral polyps, but some small patches appear to have has fully overgrown the coral's surface. Scale bars approximately 25 mm (based on size of polyps in image).

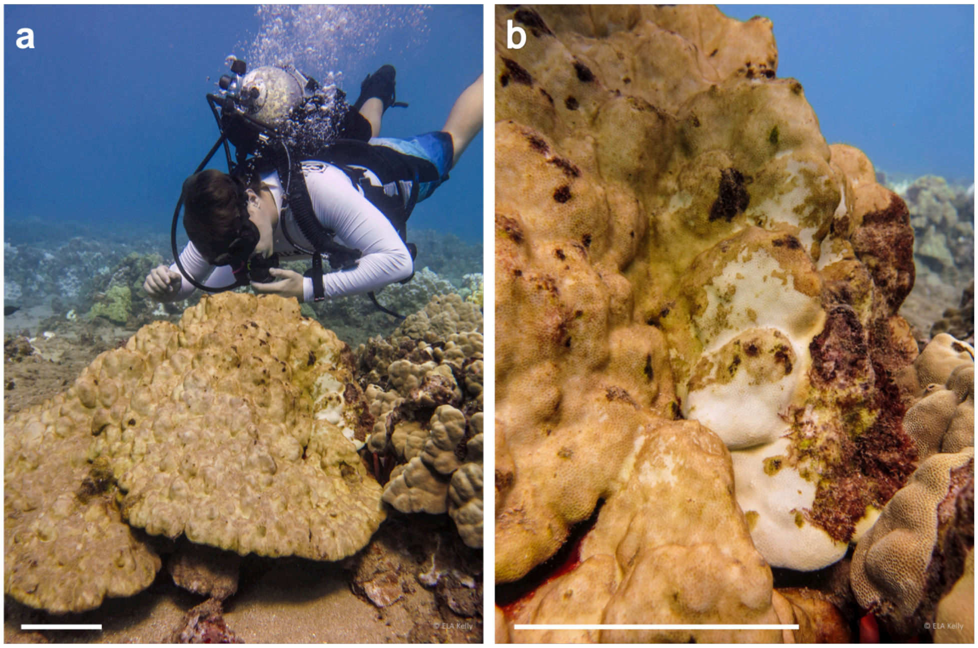

Supplementary Figure 8 | Complete overgrowth of bleached *Porites* coral by filamentous algae community. (**a-b**) Images taken with conventional underwater camera in Maui, Hawaii. At this stage the bleached coral colony has been almost completely overgrown by algae, and coral polyps fully covered by algae are dead. Scale bars, 250 mm.

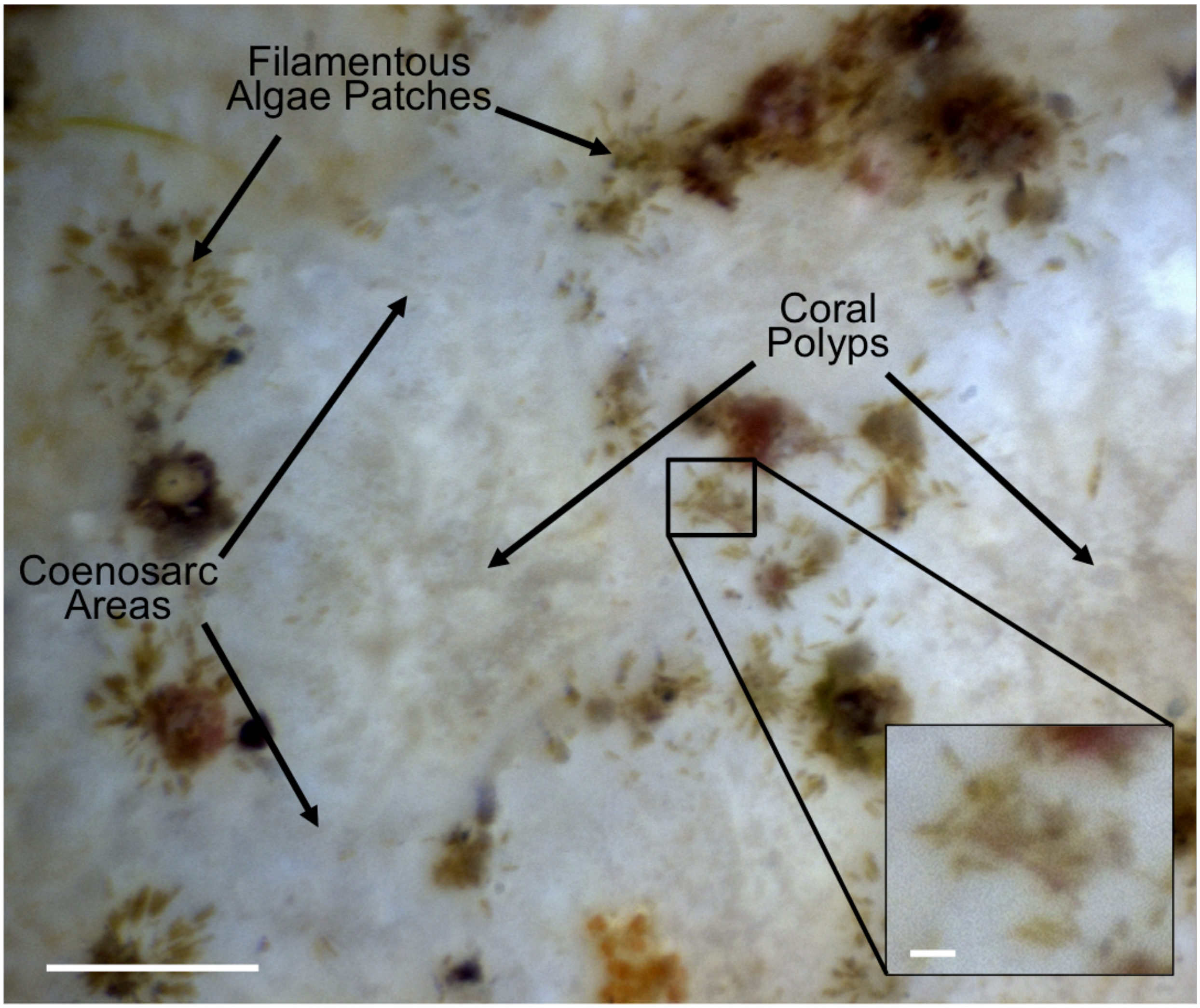

Supplementary Figure 9 | Annotated microscopy image of algal overgrowth on bleached *Porites* coral. *In situ* image acquired with the BUM at the Kahekili reef site in West Maui using the 3x objective lens. Image shows filamentous algal overgrowth on bleached *Porites lobata*. Coral polyps are bleached (and thus translucent) but still alive. If the image is closely examined the outlines of the polyp tentacles and mouth structure are visible. Filamentous algae are forming patches around the coral polyps. The area normally covered by coenosarc tissue is indicated in the image, but it is inconclusive whether or not coenosarc tissue is still intact. Main figure scale bar, 500 µm. Inset scale bar, 50 µm.

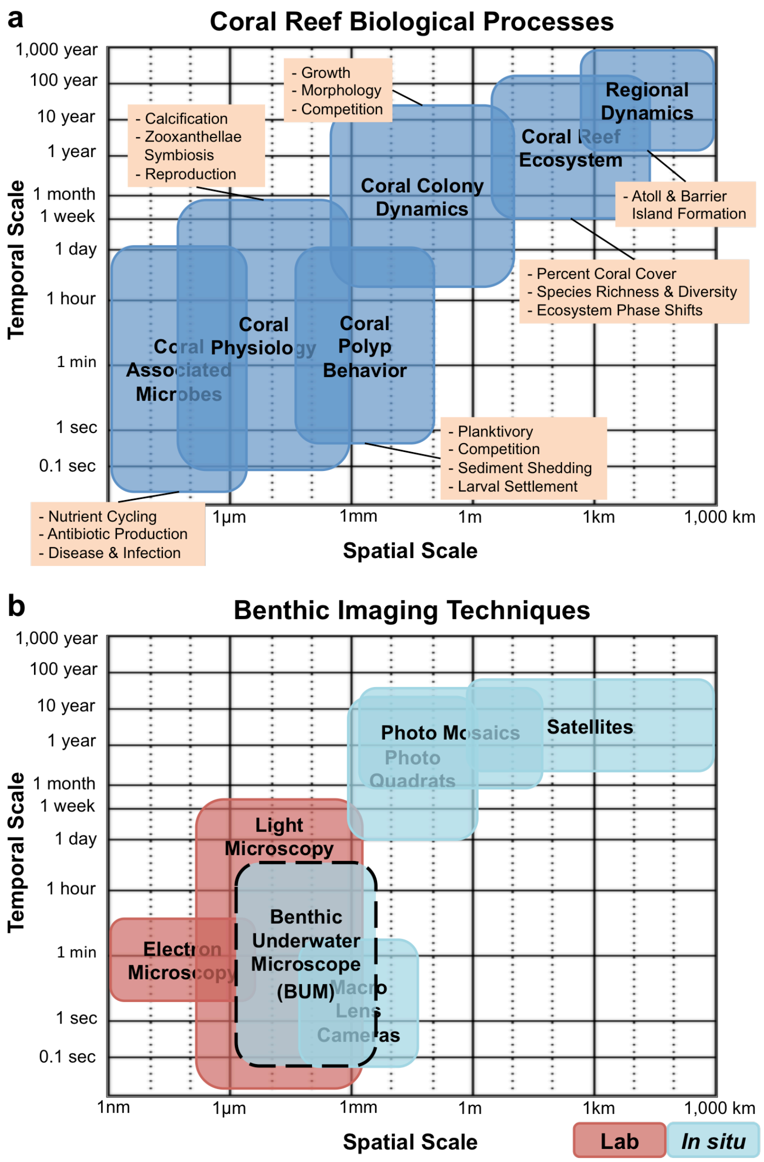

Supplementary Figure 10 | Scales of coral reef processes. Two-dimensional Stommel diagrams showing the approximate temporal and spatial scales of coral reefs processes as well as available imaging techniques. (**a**) Approximate scales associated with important coral reef biological processes. (**b**) Approximate observational scales provided by imaging techniques used in the lab and *in situ* to investigate benthic marine organisms. Note that lab microscopy techniques typically require the isolation of small coral fragments or tissues samples. Electron microscopy techniques further require specially prepared samples that are normally fixed and no longer alive, as a result the 'temporal scale' of electron microscopy is not directly comparable to other techniques. Finally, while these plots show general values, the scales examined in specific individual studies may vary from those displayed here.

Supplementary Table:

	Manufacturer Specifications		Seawater Tank Measurements with BUM			
Lens	Numerical Aperture	Working Distance in Air	Optimal Resolution	Field of View	Working Distance	Scan Range
Mitutoyo – 3x	0.09	77.0 mm	3.11 µm	2.65 mm x 2.22 mm	77.2 mm – 95.6 mm	18.4 mm
Mitutoyo – 5x	0.13	61.0 mm	2.19 µm	1.62 mm x 1.36 mm	67.8 mm – 74.7 mm	6.9 mm

Supplementary Table 1 | Instrument optical performance. Instrument optical performance as measured in the lab.
